# A Chemical Bonding Interpretation of Unusual Compressibility Trends in Hydrated Magnesium Sulfates

**DOI:** 10.1021/acs.inorgchem.5c01765

**Published:** 2025-08-26

**Authors:** Getachew G. Kebede, Ruth Franco, Fernando Izquierdo-Ruiz, Alvaro Lobato, J. Manuel Recio

**Affiliations:** † Center for Materials Science and Engineering, 16763 Addis Ababa University, Addis Ababa 1176, Ethiopia; ‡ Malta-Consolider Team and Department of Analytical and Physical Chemistry, University of Oviedo, Oviedo E-33006, Spain; § Malta-Consolider Team and Departamento de Química Física, Universidad Complutense de Madrid, Madrid E-28040, Spain

## Abstract

Hydrated magnesium sulfates (MgSO_4_·*n*H_2_O) are known to form multiple hydration states (*n* = 0–11) and are essential in planetary science and thermochemical energy storage. Despite their significance in detecting extraterrestrial water reservoirs or in mineral (de)­hydration cycles, it is still necessary to understand how the structure–property relationships of these materials evolve at different hydration levels when pressure is applied. Through a systematic first-principles computational investigation, we elucidate the key structural factors governing the densification mechanism under hydrostatic pressure of MgSO_4_·*n*H_2_O crystals. At zero pressure, we propose a useful and transferable rule of thumb that allows for straightforward evaluation of the crystal volume at any hydration level. At increasing pressure, our polyhedral and chemical bonding analyses reveal that the presence in the structure of coordinated and/or interstitial water molecules is the main factor determining the compressibility of these hydrated salts. These findings provide useful insights into the role of hydration in controlling the stability and mechanical properties of hydrated materials under extreme conditions.

## Graphical abstract

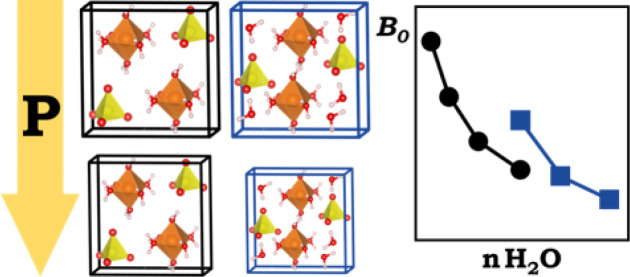

## 1 Introduction

Hydrated magnesium sulfates (MgSO_4_·*n*H_2_O) have gained significant interest in planetary and materials science research. They exist in multiple hydration states (*n* = 0–11)^1–20^ each exhibiting distinct thermodynamic stability and mechanical properties. Their ability to undergo reversible (de)­hydration reaction cycles makes them promising candidates for thermochemical energy storage.^21–24^ Furthermore, their widespread occurrence on planetary surfaces (e.g., Mars), along with other sulfate-bearing hydrated minerals such as calcium and iron sulfate hydrates, suggests their potential role in extraterrestrial water reservoirs and geochemical evolution.^25–29^ Understanding their structural stability, phase transitions, and mechanical behavior under pressure is essential for both planetary modeling and practical applications in energy storage.

Early crystallographic investigations using neutron and X-ray diffraction (ND/XRD) techniques have established the structures of various MgSO_4_ hydrates (see refs. 1–20 ), and other experimental studies examine phase stability under different external temperature^25,26,28–30^ and pressure.^4,11,17,18,31,32^ While these studies have provided valuable insights into the equation of state (EOS) of MgSO_4_·*n*H_2_O, several gaps remain. Their crystal chemistry has not been investigated in detail. The relative contributions of SO_4_ tetrahedra, MgO_6_ octahedra, and water molecules—including hydrogen-bonding networks—to the bulk modulus across different hydration states are not well understood. Additionally, previous studies^4,11,17,18,31,32^ have examined individual hydration states in isolation rather than providing a systematic analysis across the full range of compositions (*n* = 0–11), which leaves deficiencies in understanding how compressibility evolves with increasing hydration.

From a computational perspective, the atomic-scale interactions governing the stability and mechanical properties of MgSO_4_·*n*H_2_O are highly complex to describe since they involve a simultaneous combination of ionic, covalent, hydrogen bonding (H-bonds), and van der Waals forces. These competing interactions make computational modeling particularly difficult. While first-principles calculations based on density functional theory (DFT) have been increasingly employed to explore their structural and thermodynamic properties,^33–35^ systematic studies on the EOS of MgSO_4_·*n*H_2_O across different hydration states remain scarce.

To address these concerns, we employ state-of-the-art DFT calculations and chemical bonding theoretical tools to systematically investigate the EOS of MgSO_4_·*n*H_2_O in a hydrostatic pressure range of up to 10 GPa. By analyzing energy-volume (*E*-*V*) and pressure–volume (*p*-*V*) relationships, along with a detailed polyhedral decomposition in terms of MgO_6_ octahedra, SO_4_ tetrahedra, and water-filled voids, we aim to clarify their relative contributions to compressibility. These analyses are extended to the chemical bonding network using the QTAIM formalism^36^ to provide chemical insights into the densification mechanisms that control the volume reduction of hydrated salts under pressure. As the space associated with water is the most compressible, a clear distinction is made between interstitial water molecules and those that are directly coordinated with Mg. This thorough study allows us to provide general trends that could be transferable to other sulfate salts or similar hydrated minerals.

By providing a comprehensive EOS characterization of MgSO_4_·*n*H_2_O across multiple hydration states, our study offers a first step with new chemical insights into the fundamental mechanisms controlling the mechanical behavior of hydrated sulfates. These findings have broad implications for planetary science, thermochemical energy storage, and the predictive modeling of hydrated salt compressibility under extreme conditions. More steps forward along the explicit consideration of the stability of these salts under varying temperature and pressure conditions are currently in preparation in a separate manuscript.

## 2 Computational Details

### 2.1 Structural Description

We selected nine MgSO_4_·*n*H_2_O systems with *n* = 0, 1, 3, 4, 5, 6, 7, 9, and 11, where their crystallographic information is available in the literature. The space group and the structure references used for the starting DFT input geometries are shown in bold in Table 1. All the calculations were performed using conventional unit cells, except for the cases of *n* = 3 and 6. For MgSO_4_·6H_2_O, a primitive unit cell was employed, consisting of 96 atoms. In MgSO_4_·3H_2_O, one of the H atoms is dynamically disordered between two sites (fractional occupations of 0.57 and 0.43).^5^ We employed a 2 × 1 × 1 supercell consisting of 240 atoms (*Z* = 16) to investigate MgSO_4_·3H_2_O. In the MgSO_4_ hydrates with *n*= 1.25, 2, 2.4, 2.5, and 4 (specifically cranswickite), the positions of H atoms were not reported in the XRD/ND measurements^6,7,9,27^ and thus, are not considered in this study.

**Table 1:** Hydrated Mg-Sulfates Studied in This Work together with Their Space Group (SG) and Experimental References from which Our Starting Structures Were Taken (in Bold)

Phase	SG	References
α-MgSO_4_	*Cmcm*	Rentzeperis and Soldatos,^1^ **Fortes et al**.^2^
MgSO_4_·1H_2_O	*C*2/*c*	**Hawthorne et al**.,^3^ Meusburger et al.^4^
MgSO_4_·3H_2_O	*Pbca*	**Fortes and Lemée-Cailleau** ^5^
MgSO_4_·4H_2_O	*P*2_1_/*n*	Baur,^8^ C. Peterson^9^
MgSO_4_·5H_2_O	*P1̅*	Baur and Rolin,^10^
MgSO_4_·6H_2_O	*C*2/*c*	Zalkin et al.,^12^ **Batsanov** ^13^
MgSO_4_·7H_2_O	*P*2_1_2_1_2_1_	Baur et al.,^14^ Ferraris et.,^15^ Fortes **et al.**,^16^ Gromnitskaya et al.^17^
MgSO_4_·9H_2_O	*P*2_1_	**Fortes et al**.^18^
MgSO_4_·11H_2_O	*P1̅*	Peterson al.,^19^ **Fortes et al**.^20^

Hydrates with *n* = 0, 3, 4, and 7 crystallize in orthorhombic crystal structures, while those with *n* = 1, 5, 6, and 9 crystallize in monoclinic structures. The highest hydration state, *i.e., n* = 11 crystallizes in a triclinic structure. The crystal structure and polyhedral connectivity views of selected magnesium sulfate hydrates are illustrated in Figure 1 using the VESTA utility.^37^ The remaining unit cells are depicted in Figure S1. The building blocks of all of these structures are SO_4_ tetrahedra and MgO_6_ octahedra.

**Figure 1:**
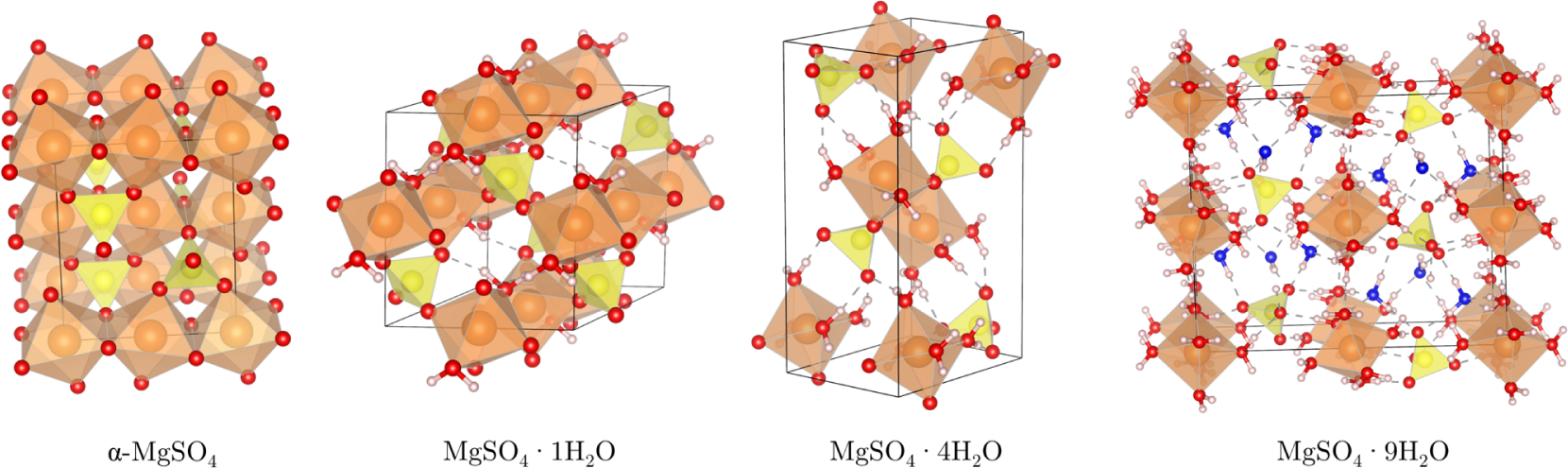
Polyhedral views of the unit cells of the MgSO_4_·*n*H_2_O with *n* = 0, 1, 4, and 9. Atoms: Mg = orange inside MgO_6_ octahedra, S = yellow inside SO_4_ tetrahedra, H = gray, and O = red when coordinated to sulfur and for noninterstitial water, while interstitial water oxygen atoms are denoted in blue.

In anhydrous α-MgSO_4_, the Mg^2+^ ion is coordinated to six oxygen atoms from the SO_4_ groups, forming both corner- and edge-sharing MgO_6_ octahedra, which result in two inequivalent Mg–O bond lengths. Two Mg–O bonds participate in corner-sharing, while the remaining four are involved in edge-sharing, with the latter being slightly longer. In the MgSO_4_·1H_2_O structure, two water oxygen atoms are coordinated to MgO_6_ polyhedra, and the four oxygen atoms of the SO_4_ group form corner-sharing coordination with MgO_6_ polyhedra. As *n* increases, the direct polyhedral connectivity between SO_4_ and MgO_6_ decreases. In the MgSO_4_·4H_2_O structure, the four corners of the MgO_6_ octahedra are occupied by the oxygen atoms from water molecules, and two of the oxygen atoms of SO_4_ are bound to the MgO_6_ octahedra, while the other two oxygen atoms of SO_4_ are detached from the octahedra and are involved in accepting three hydrogen bonds from water molecules. Beyond *n* = 6, MgO_6_ and SO_4_ are not directly coordinated by water molecules. The additional water molecules beyond *n* = 6 occupy interstitial sites. The blue oxygen atoms of MgSO_4_·9H_2_O in Figure 1 represent interstitial water molecules.

### 2.2 Details of DFT Calculations

While a thorough investigation of the performance of van der Waals (vdW) exchange-correlation (XC) functionals for equations of state (EOS) may require an independent study, it has been generally observed that vdW-XC functionals tend to provide good predictions of hydrate structures, including equilibrium H-bond distances and cell volumes, in comparison to traditional GGA or LDA approaches.^34^ In this study, we first assessed the performance of several vdW-XC functionals by comparing the lattice parameters and unit cell volumes of MgSO_4_·*n*H_2_O (*n* = 0–11) calculated using four functionals: PBE,^38^ PBE-D3,^39^ reV-vdW-DF2,^40^ and optPBE-vdW.^41^ The results were benchmarked against experimental crystallographic data in Table S1.

The PBE functional consistently overestimated cell volumes with average deviations of 4.9%. Inclusion of dispersion corrections in PBE-D3 and optPBE-vdW improved agreement, reducing the mean volume errors by approximately 2.1–2.5%. Among the tested functionals, reV-vdW-DF2 showed the best overall performance, with the lowest mean absolute percentage errors in both lattice constants and volumes (1.7%). Based on this, we selected reV-vdW-DF2 for the EOS calculations in this work. This choice aligns with prior observations reported in the literature, which provide evidence of the capacity of this functional for describing hydrogen bonds.^34^ We expect the bulk moduli obtained using vdW functionals to lie slightly above those predicted by PBE and below those from LDA.

The DFT calculations were conducted using the VASP program.^42–45^ The electron–core interactions were described using the PAW formalism.^45^ For Mg, S, O, and H, the 2p^6^3s^2^, 3s^2^3p^4^, 2s^2^2p^4^, and 1s^1^ electrons, respectively, constitute the valence active space. We tested the convergence of *k*-points with 0.15, 0.20, 0.25, and 0.30 Å^–1^ distances, and the results presented were performed with 0.20 Å^–1^ *k*-point distances. All the calculations were performed with a plane-wave cutoff of 600 eV. Except for the disordered MgSO_4_·3H_2_O, all of the structures were optimized until the force on each atom was less than 0.005 eV/Å. The supercell structure MgSO_4_·3H_2_O was optimized until the forces were less than 0.01 eV/Å.

### 2.3 Gibbs EOS Fittings

The structures obtained from the references quoted in Table 1 are expanded and contracted by up to 20% around the vicinity of the equilibrium cell volume. A homogeneous grid of 20 volumes within this cell volume range was selected. A volume-constrained structural optimization was performed for each *V*, allowing the atomic coordinates to be relaxed. The zero temperature and zero pressure equilibrium volume (*V*_0_), electronic energy (*E*_0_), bulk modulus (*B*_0_), and its first pressure derivative were obtained by fitting the Vinet EOS^46^ to the calculated (*E*_*i*_,*V*_*i*_) data points. This procedure allows us to calculate *p*-*V* curves using our in-house-developed Gibbs code.^47,48^

### 2.4 Polyhedral Partition of EOS Parameters

The formula unit cell volume (*Z* = 1) of the hydrated salts can be exhaustively partitioned in terms of disjoint regions (*V*_*p*_) associated with SO_4_ tetrahedra $\left(V_{{\mathrm{SO}}_{4}}\right)$ , MgO_6_ octahedra $\left(V_{{\mathrm{MgO}}_{6}}\right)$ , and the remaining polyhedral space occupied by water molecules (*V*_w_): *V* = ∑*V*_*p*_, *p* = SO_4_, MgO_6_, w. By defining local polyhedral compressibilities (*k*_*p*_) at zero pressure as^49,50^

1 $$k_{p}=-\frac{1}{V_{p}}\frac{\partial V_{p}}{\partial p}$$

an expression for the zero-pressure bulk compressibility (*k*_0_) of any of the hydrated salts can be straightforwardly derived in terms of these local contributions:

2 $$k_{0}=\sum f_{p}k_{p};\;f_{p}=\frac{V_{p}}{V_{0}}$$

where *f*_*p*_ are the fractional occupations of the polyhedra and the remaining space within the unit cell, and *V*_0_ is the crystal volume per formula unit, all evaluated at zero pressure. The local contributions to the zero-pressure bulk modulus (*B*_0_) are implicitly included in $k_{0}=\frac{1}{B_{0}}$ .

## 3 Results and Discussion

### 3.1 *E*-*V* and *p*-*V* Curves for Anhydrous and Hydrated Magnesium Sulfate Salts

The calculated set of *E*(*V*) points and the corresponding *p*(*V*) curves for MgSO_4_·*n*H_2_O (*n* = 0, 1, 3, 4, 5, 6, 7, 9, 11) systems are plotted in Figure 2. EOS parameters are gathered in Table 2. It is evident from Figure 2 that the relationship between *V*/*V*_0_ and pressure follows an overall systematic pattern with the degree of hydration (*n*). Anhydrous MgSO_4_ turns out to be the least compressible member among the MgSO_4_·*n*H_2_O series, whereas MgSO_4_·11H_2_O is the most compressible hydrate. The compressibilities of the intermediate compositions fall between these two extremes. As *n* increases, the structures exhibit greater flexibility with few exceptions. In highly hydrated materials, the prevalence of H-bonds and the relatively loose packing of the ionic entities (Mg^2+^ and ${\mathrm{SO}}_{4}^{2-}$ ) contribute to their high compressibility.

**Figure 2:**
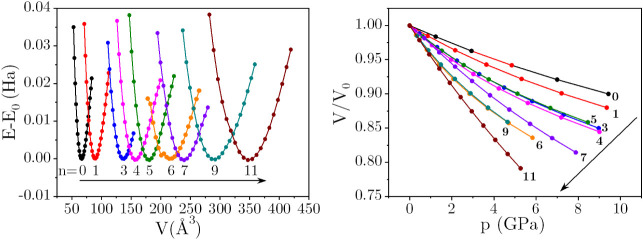
(Left) Calculated *E*(*V*) curves for MgSO_4_·*n*H_2_O. Each hydrate’s energy is shifted relative to its energy calculated at *V*_0_, for ease of comparison. (Right) Normalized volume versus pressure plots derived from the corresponding *E*(*V*) curve.

**Table 2:** Calculated EOS Parameters, Including the Formula Unit Equilibrium Volume (*V*_0_), Bulk Modulus (*B*_0_), and Its First Pressure Derivative $\left(B_{0}^{\prime }\right)$ , All Evaluated at Zero Pressure for MgSO_4_·*n*H_2_O

MgSO_4_·*n*H_2_O	*V*_0_(Å^3^)	*B*_0_(GPa)	$B_{0}^{\prime }$ (GPa)
α-MgSO_4_	66.55	69.17	5.17
MgSO_4_·1H_2_O	89.23	53.38	5.00
MgSO_4_·3H_2_O	136.78	37.58	4.72
MgSO_4_·4H_2_O	157.30	33.40	5.65
MgSO_4_·5H_2_O	179.97	36.52	5.57
MgSO_4_·6H_2_O	213.34	20.66	5.18
MgSO_4_·7H_2_O	238.83	28.17	4.19
MgSO_4_·9H_2_O	290.12	22.21	4.28
MgSO_4_·11H_2_O	348.43	21.37	2.41

### 3.2 *V*_0_-*n* and *B*_0_-*n* Curves for Anhydrous and Hydrated Magnesium Sulfate Salts

The obtained EOS parameters, namely the formula unit equilibrium volume (*V*_0_), bulk modulus (*B*_0_), and its pressure derivative $\left(B_{0}^{\prime }\right)$ , all evaluated at zero pressure, for the hydrated MgSO_4_ family considered in this work are presented in Table 2 and plotted in Figure 3. The *B*_0_ of the MgSO_4_·*n*H_2_O family is provided on the right axis in Figure 3 while the *V*_0_ of the MgSO_4_·*n*H_2_O is given on the left axis. The black data points in Figure 3 show that there is a quasilinear correlation between *V*_0_ and the hydration state (*n*). The volume increases smoothly from 66.55 Å^3^ (for *n* = 0, anhydrous MgSO_4_) to 348.43 Å^3^ (for *n* = 11, the highest water content). Based on the calculated (*n*, *V*_0_) data points, a simple linear regression model can be proposed to predict the volume of a given hydrate using the known value of *V*_0_ for anhydrous MgSO_4_. The equation is as follows: *V*_0_(MgSO_4_·*n*H_2_O) = 62.677 + 25.270*n*, with *R* ^2^ = 0.997.

**Figure 3:**
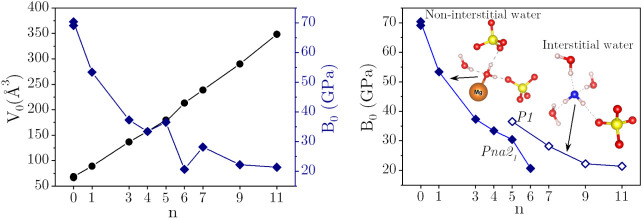
(Left) *V*_0_ (black circles) and *B*_0_ (blue squares) evolution with the number of water molecules in the MgSO_4_·*n*H_2_O crystal family. (Right) The same as the (*n*,*B*_0_) data points in the left panel, but here, hydrates containing interstitial water (open blue squares) and hydrates with no interstitial water molecules (filled blue squares) are plotted separately to highlight that they follow systematically different patterns. Note that the two points appearing at *n* = 0 and *n* = 5 correspond to two different polymorphs of these systems. The inset schematics provide examples of interstitial and noninterstitial water molecules. Color code: orange = Mg (extra-large to highlight the coordination with the noninterstitial water molecule), white = H, yellow = S, red = O, and blue = O from the interstitial water molecule.

In their volume-based thermodynamics formalism, Glasser and Jenkins extensively studied the volume variation with *n* in hydrates.^51–53^ They found a linear relation between *n* and the volume difference between hydrate and anhydrate. Using this relation, they predicted a water volume of 24.5Å^3^. The slope obtained in our fitting is in excellent agreement with this value and allows us to interpret the increasing volume of the hydrated salts as independent of the anhydrous sublattice, which seems to keep the same volume regardless of the number of water molecules incorporated into the unit cell. We believe that this result could be inter- or extrapolated to sulfate hydrates containing different water molecules. It also encourages us to use it as a rule of thumb to predict the volume of other hydrated salts given only the volume of the corresponding anhydrate.

From the known inverse *B*_0_-*V*_0_ relationship,^54^ and given the linear *n*-*V*_0_ correlation, we would expect a continuous decreasing trend of *B*_0_ with *n* increasing. The variation of *B*_0_ with *n* is illustrated in Figure 3 (blue squares). Although the variation of *B*_0_ with *n* shows some kinks and is not strictly monotonic, it is apparent that, as the number of water molecules per MgSO_4_ unit increases, *B*_0_ decreases from its maximum value of 69.17 GPa for anhydrous MgSO_4_ toward a relatively constant value around 20 GPa. While our calculated *B*_0_ values are slightly higher than the experimental ones (MgSO_4_·1H_2_O: 48.1 ± 5 GPa,^4^ MgSO_4_·7H_2_O: 21.5 ± 5 GPa,^16^ MgSO_4_·9H_2_O: 19.95 ± 3 GPa^18^ and MgSO_4_·11H_2_O:19.9 ± 4 GPa,^32^) the trend of MgSO_4_·*n*H_2_O becoming more compressible as *n* increases agrees with experimental observations.

However, despite previous efforts to implement volume-based thermodynamics for predicting compressibilities of common minerals,^55^ our results show the need for microscopic structural details to accurately predict bulk moduli, even for hydrates with similar compositions. Comparing the *V*_0_ *vs n* and *B*_0_ *vs n* relations, it is clear that the kinks in the *B*_0_ trend do not align with the inverse *B*_0_ - *V*_0_ relationship, which prevents a simple model for *B*_0_, as we have found for *V*_0_, and requires further explanation.

In order to uncover the existence of different patterns governing the evolution of *B*_0_ with the hydration degree, we have reexamined the (*n*, *B*_0_) data points in Figure 3 (right). Notably, hydrates with *n* = 0, 1, 3, 4, and 6 follow the same curve (filled blue squares), displaying rapid variation. Conversely, compositions with *n* = 5, 7, 9, and 11 follow a different curve (open blue squares), showing a minor decrease in *B*_0_ (e.g., 22.21 GPa for *n* = 9 and 21.37 GPa for *n* = 11). Upon detailed crystal structural inspections, we found that the underlying difference between these curves may be linked to the presence or absence of noncoordinated interstitial water molecules adjacent to Mg^2+^. In fact, the water molecules contained in the MgSO_4_·*n*H_2_O unit cell can be classified as either (i) noninterstitial, coordinated to Mg^2+^, or (ii) interstitial water molecules, not directly bonded to Mg^2+^.

For hydrated Mg sulfates with *n* = 5, 7, 9, and 11, the number of interstitial water molecules is 1, 1, 3, and 5, respectively (see Figures 1 and S1). These interstitial water molecules accept two H-bonds from neighboring water molecules and donate two H-bonds to sulfate oxygen(s) and other water molecules. The Mg octahedra and sulfate tetrahedra are connected through these interstitial water molecules. In hydrates with *n* = 1, 3, 4, and 6, there are no interstitial water molecules.

The presence or absence of interstitial water molecules in the structure gives rise to different compressibility patterns, as shown in Figure 3 (right). This is due to changes in both the volume effect introduced by the water molecules and the water–salt interactions. A key observation is that the compressibility of hydrates with a given composition is primarily governed by hydrogen bond interactions between structural units and interstitial water molecules.

To further analyze the difference between interstitial and coordinated water molecules, we calculated the Bader charges of MgSO_4_·7H_2_O, where six molecules are classified as “coordinated water,” and the seventh water molecule is “interstitial water.” The results are shown in Figure 4. As expected, the sulfur-bound oxygen atoms are more negative, with a charge of −1.36|*e*|. The oxygen of the coordinated water molecules exhibits values ranging from −1.269 to −1.283|*e*|, which contrasts with the higher value of −1.234|*e*| for the oxygen of the interstitial water molecule (represented by the blue oxygen atom). Although this difference in charge is not significant, it is sufficient to demonstrate the distinct roles of interstitial and coordinated water molecules in influencing the structural and mechanical properties of hydrated magnesium sulfates.

**Figure 4:**
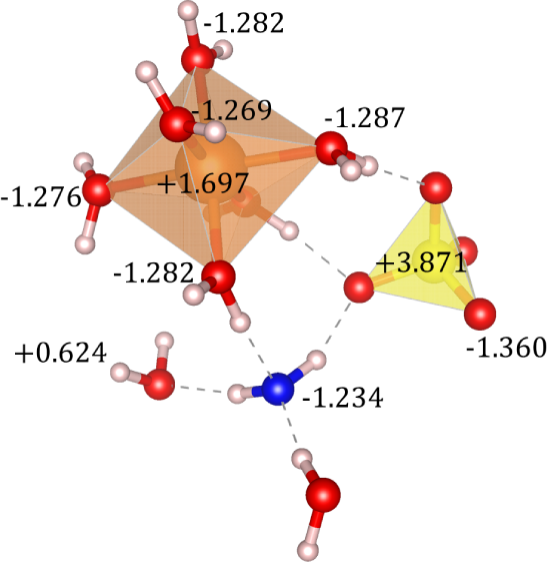
Bader charge analysis of atoms in MgSO_4_·7H_2_O, highlighting the differences between coordinated and interstitial water molecules. Atoms: Mg = orange inside MgO_6_ octahedra, S = yellow inside SO_4_ tetrahedra, H = gray, and O = red when coordinated to sulfur and for noninterstitial water, while the interstitial water oxygen atom is denoted in blue.

At this stage, it is important to highlight an interesting case concerning magnesium sulfate pentahydrate, which exists in two distinct polymorphs: (i) an ambient-pressure phase that crystallizes in the *P1* space group,^10^ and (ii) a high-pressure dehydration product of MgSO_4_·7H_2_O, identified by Wang et al.,^11^ which crystallizes in the *Pna2*_*1*_ space group.

The *Pna2*_*1*_ polymorph is characterized by isolated, corner-linked MgO_6_ and SO_4_ polyhedra, in contrast to the infinite chains of corner-linked MgO_6_ and SO_4_ polyhedra found in the ambient-pressure *P1* polymorph. A key structural difference between these two phases is that, in the *Pna2*_*1*_ phase, all five water molecules are directly coordinated to the Mg^2+^ ion, whereas the *P1* polymorph contains one interstitial water molecule in addition to four coordinated water molecules.

Our calculations of *B*_0_ for the *Pna2*_*1*_ phase yield a value of 31.78 GPa, which closely follows the trend observed for the hydrates with noninterstitial water molecules (see the solid-line curve with filled blue circles in Figure 3 right). This value differs from that found for the *P1* phase with one interstitial water molecule (36.52 GPa), further reinforcing the correlation between the bulk modulus and hydration number, particularly with regard to the presence or absence of interstitial water molecules. This relationship plays a critical role in determining the compressibility of hydrated magnesium sulfate phases and provides valuable insights into their mechanical stability under varying pressure conditions.

Finally, it is noteworthy that MgSO_4_·6H_2_O is more compressible than MgSO_4_·7H_2_O. In the structure of MgSO_4_·6H_2_O, all the water molecules are coordinated to Mg, and there are no water molecules that act as a scaffold for structural support. This makes MgSO_4_·6H_2_O more compressible.

### 3.3 Crystal Chemistry of Magnesium–Sulfate Hydrates

In this section, we will elucidate the variation of the compressibilities with the hydration state in terms of structural unit (polyhedra) connectivities. Calculated data corresponding to the polyhedral decomposition of eq 2 are collected in Table 3. Initially, we verified that the *B*_0_ bulk values presented in Table 2 are recovered from the sum of the polyhedral compressibilities using eq 2. The comparison of the calculated *B*_0_ value using eq 2 with *B*_0_ in Table 2 is very satisfactory, as it yields differences lower than 1 GPa. Next, we examine the dependence on *n* of the particular EOS parameters (*V*_0_ and *B*_0_) calculated for SO_4_ tetrahedra and MgO_6_ octahedra (see Figure S2) and for the remaining voids filled with water molecules (see Figure 5).

**Table 3:** Calculated Fractional Occupations and Polyhedral Compressibilities (See eqs 1 and 2)­^a^

Phase	$f_{SO_{4}}$	$k_{SO_{4}}$	$f_{MgO_{6}}$	$k_{MgO_{6}}$	*f*_w_	*k*_w_	*k*_0_	*B*_0_	Δ*B*_0_
α-MgSO_4_	0.025	0.610	0.183	0.231	0.793	0.012	0.014	71.30	–0.95
MgSO_4_·1H_2_O	0.019	0.480	0.134	0.201	0.847	0.017	0.019	53.36	0.02
MgSO_4_·3H_2_O	0.012	0.346	0.086	0.124	0.902	0.026	0.027	37.29	–0.01
MgSO_4_·4H_2_O	0.011	0.363	0.076	0.101	0.913	0.029	0.030	33.44	–0.04
MgSO_4_·5H_2_O	0.009	0.387	0.066	0.104	0.924	0.026	0.027	36.54	–0.02
MgSO_4_·6H_2_O	0.008	0.318	0.056	0.065	0.936	0.050	0.052	19.73	0.93
MgSO_4_·7H_2_O	0.007	0.277	0.050	0.078	0.943	0.035	0.035	28.18	–0.01
MgSO_4_·9H_2_O	0.006	0.274	0.041	0.070	0.952	0.044	0.045	22.12	0.09
MgSO_4_·11H_2_O	0.005	0.272	0.034	0.057	0.961	0.045	0.047	21.32	0.05

a For *n* = 5, 6, 9, and 11, the MgO_6_ octahedral values represent the average of the two symmetry-inequivalent MgO_6_ octahedra. Units are given in GPa^–1^ × 10^–4^, GPa^–1^ × 10^–2^, GPa^–1^, and GPa^–1^ for the compressibilities of SO_4_, MgO_6_, water (w), and the bulk, respectively. The last column presents the difference between the calculated *B*_0_ in GPa obtained using eq 2 and *B*_0_ presented in Table 2.

**Figure 5:**
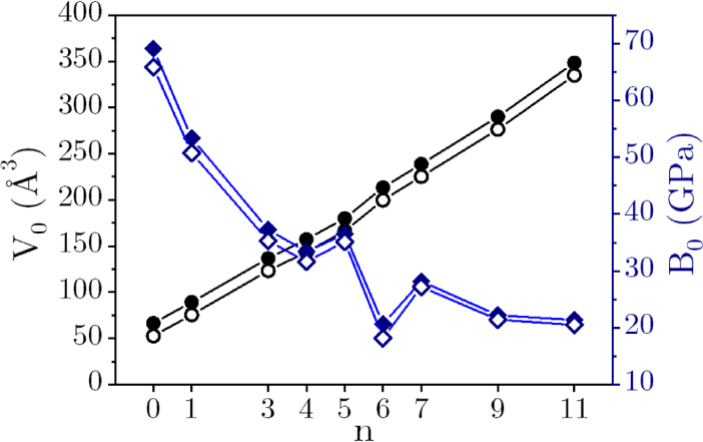
Variation of the water-filled and void space volumes, *V*_0_ (black open circles) and *B*_0_ (blue open squares), as a function of the number of water molecules in hydrated Mg-sulfate materials. The corresponding bulk crystal volumes, *V*_0_ (black filled circles) and *B*_0_ (blue filled squares), are plotted for comparison. The results indicate that the bulk compressibility primarily originates from the water-filled and void spaces.

The volume of SO_4_ shows a very marginal dependence on *n*. It varies between 1.65 and 1.73 Å^3^ as shown in Figure S2. However, this is not the case for the *B*_0_ of SO_4_; it decreases from 425 to 166 GPa as *n* changes from 0 to 11. The *B*_0_ of SO_4_ in MgSO_4_·1H_2_O is 414 GPa, which is lower than that of the anhydrate (425 GPa). This demonstrates that SO_4_ gradually becomes more compressible as *n* increases. However, it is important to note that although the *B*_0_ of SO_4_ undergoes significant changes with *n* (variation around 61% from anhydrous to *n* = 11), its contribution to the bulk modulus of the crystal is not substantial. This is attributed to the fact that the occupation fraction of SO_4_, $f_{SO_{4}}$ , is very small and decreases as *n* increases (see eq 2). For instance, $f_{{\mathrm{SO}}_{4}}=2.5\times 10^{-2}$ for *n* = 0, and $f_{{\mathrm{SO}}_{4}}=0.5\times 10^{-3}$ for *n* = 11. As a result, the contribution of SO_4_ to the bulk compressibility remains nearly constant and small across the entire MgSO_4_·*n*H_2_O series. Similarly, MgO_6_ octahedra show a marginal contribution to the bulk compressibility in spite of presenting *B*_0_ values between 50 and 80 GPa. The reason again is the low values of the occupation factor, $f_{{\mathrm{MgO}}_{6}}$ , below 0.2 for all hydration degrees.

The variations with *n* of volumes and bulk moduli of the remaining space not occupied by SO_4_ and MgO_6_ polyhedra are key to understand the compression mechanism of these hydrates (see Figure 5). As *n* increases, *V*_w_ becomes much greater (varies between 50–330 Å^3^) as the additional volume of water is included in the voids in our partition of the unit cell space. Similar to Figure 3, we argue that water–water interactions play a much greater role in the compressibility of MgSO_4_·nH_2_O than do SO_4_ and MgO_6_ do. When the MgSO_4_·nH_2_O systems considered are compressed, they mainly do so by compressing the H-bond networks and the empty space; this directly proves that higher hydration states tend to be more compressible.

The polyhedral decomposition of the unit cell volume and compressibility provides support for our findings. The SO_4_ and MgO_6_ contributions to volume and compressibility are not significant. It is the water-filled space that dominates the volume and, more importantly, accounts for more than 90% of the value of the bulk compressibility. As a result, the same expected and nonexpected trends earlier seen for the unit cell volume and the bulk modulus are repeated again when *V*_w_ and *B*_0, w_ from the water region are evaluated. This is clearly illustrated in Figure 5.

### 3.4 Bonding Compressibilities

A step forward is given here in analyzing the effects of pressure on S–O, Mg–O, and hydrogen bond lengths. This type of analysis has proven crucial in highlighting the mechanisms of phase transitions in similar hydrated salts (see, for example, ref. 56). For this purpose, we selected MgSO_4_·*n*H_2_O with *n* = 0, 1, 4, and 7. Anhydrous α-MgSO_4_ (*n* = 0) serves as the baseline, where Mg^2+^ and ${\mathrm{SO}}_{4}^{2-}$ form a rigid framework without hydrogen bonds. The monohydrate (*n* = 1) introduces water molecules that participate in hydrogen bonding while maintaining a direct Mg–O coordination. The tetrahydrate (*n* = 4) constitutes an intermediate scenario, where a significant portion of sulfate oxygen atoms are no longer bonded to Mg, leading to a transition toward water-mediated coordination. The heptahydrate (*n* = 7) represents the fully hydrated phase, in which sulfate groups and Mg^2+^ ions are entirely coordinated via water molecules with one interstitial molecule, resulting in an extensive hydrogen bond network. We present in Figure 6 the variations in S–O and Mg–O bond lengths under pressure in these four representative Mg-sulfate hydrates. Solid and dashed lines represent distances in the SO_4_ tetrahedra and MgO_6_ octahedra, respectively. The coordination environments of SO_4_ and MgO_6_ are depicted in the corresponding insets of Figure 6.

**Figure 6:**
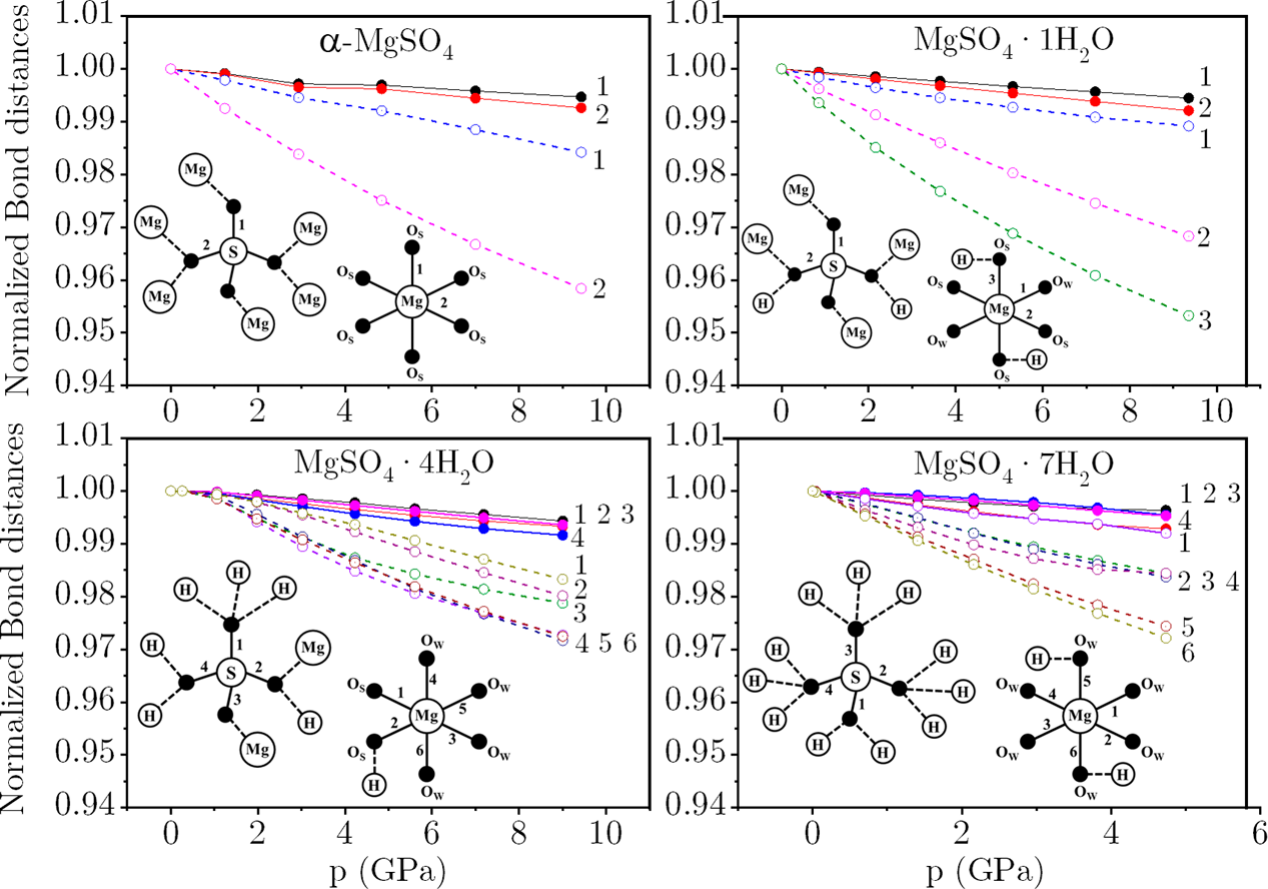
Pressure dependence of S–O bond distances (solid lines) and Mg–O bond distances (dashed lines) in the SO_4_ and MgO_6_ polyhedra for *n* = 0, 1, 4, and 7. The insets in each panel illustrate the SO_4_ and MgO_6_ bonding environments within the bulk crystal structure. Numbers at the end of each line correspond to the specific bonding configurations of SO_4_ and MgO_6_ shown in the respective insets.

In α-MgSO_4_ (top-left panel of Figure 6), two of the oxygen atoms in SO_4_ participate in corner-sharing with MgO_6_ octahedra, while the other two are involved in edge-sharing between MgO_6_ units (see also Figure 1). At ambient pressure (*p* = 0), the respective S–O bond distances are 1.463 and 1.511 Å. These bonds exhibit minimal compressibility under pressure, with the S–O bonds involved in MgO_6_ edge sharing (red solid line) being slightly more compressible than the corner-sharing ones (black solid line), which reflects that the greater the distance, the greater the compressibility. Similarly, two distinct Mg–O bonds exist in MgO_6_: bonds involving O atoms bridging Mg and S (blue dashed line) and Mg–O bonds where O atoms are connecting MgO_6_ octahedra through the edges (pink dashed line). At *p* = 0, their respective bond distances are 2.017 and 2.144 Å. Again, the edge-sharing Mg–O bonds are more compressible than the shorter corner-sharing ones.

As expected, due to the lower positive charge of Mg, Mg–O bonds exhibit greater compressibility than S–O bonds, and the disparity in compressibility among Mg–O bond groups is larger than that observed for S–O bonds. An electron density analysis of the S–O and Mg–O bonds reveals a similar conclusion. S–O bonds present, on average, an electron density value at the bond critical point almost 10 times greater (ρ ≈ 0.30 au) than that for Mg–O bonds (ρ ≈ 0.04 au). This greater value of the electron density is associated with the covalent contribution of the S–O bonds, as evidenced by the negative values of the Laplacian of the electron density. In contrast, Mg–O bonds always present a positive Laplacian value, as expected from their marked ionic character (see Table S3). This behavior underscores the higher rigidity of the SO_4_ tetrahedra compared to that of MgO_6_, consistent with the polyhedral discussion above.

The insets in Figure 6 illustrate the progressive changes in the coordination environments of SO_4_ and MgO_6_ upon the incorporation of water into the MgSO_4_ crystal. The strength of the MgO_6_···SO_4_ linkage starts to weaken, with Mg coordinating to water oxygen atoms, while the SO_4_ oxygen atoms form hydrogen bonds with water molecules. In MgSO_4_·1H_2_O, two of the sulfate oxygens are bicoordinated to both H and Mg^2+^, while the remaining two oxygens are singly bonded to Mg^2+^. In MgO_6_, all six oxygen atoms participate in corner-sharing, with two oxygens originating from water molecules and the remaining four from SO_4_. In MgSO_4_·4H_2_O, two sulfate oxygen atoms are detached from Mg, while the remaining two are bonded to one Mg atom and a hydrogen bond. The corners of the MgO_6_ octahedra are occupied by four water molecules and two sulfate oxygen atoms. In MgSO_4_·7H_2_O, all sulfate oxygen atoms are detached from Mg, and both SO_4_ and MgO_6_ are coordinated exclusively with water molecules through hydrogen bonding and direct coordination with water oxygens.

The variation of the S–O bond distances with pressure does not show significant changes across different hydration levels. Despite the different coordination environments of the SO_4_ tetrahedra with increasing *n*, the compressibility of the S–O bond remains nearly constant. This fact can be traced back to the nature of these bonds, which, according to our electron density analysis, barely change regardless of the hydration number (Table S3). The compressibility of Mg–O bonds becomes increasingly isotropic as *n* increases from 1 to 4 to 7, but with a similar average value independent of the hydratation degree. These observations highlight the inherent rigidity of SO_4_ and MgO_6_, as reflected in the limited compressibility of the S–O and Mg–O bonds. It is worth noting that the normalized bond lengths are plotted within a narrow range (0.94–1.00) and that most of the length reductions are less than 3% in the 0–10 GPa pressure range. As discussed below, the most significant structural changes under pressure are observed in the geometry of the H-bond network.

To examine the effects of pressure on these H-bonds in the MgSO_4_·*n*H_2_O crystal family, we analyzed variations in well-recognized geometric parameters of H-bonds, namely the intramolecular O–H bond length (*R*(O–H)), donor–acceptor intermolecular distances (*R*(O···O) and *R*(H···O)), H-bond angle (∠(O–H···O)), and the correlation between these parameters under compression. In MgSO_4_·1H_2_O two equivalent moderately strong O_w_-H···O_S_ H-bonds exist between the water molecule (O_w_) and the SO_4_ unit (O_S_). Moderately means that the strength is lower than that of the covalent O–H bond but higher than weak van der Waals interactions. The distances and angles calculated for the donor–acceptor H-bond at ambient pressure lie within a range of typical values: *R*(O_w_–H) = 1.005 Å, *R*(H···O_S_) = 1.741 Å, and ∠(O_w_-H ··· O_S_) = 160^◦^. The expected interplay between the covalent *R*(O_w_–H) and H-bonding *R*(H···O_S_) distances upon compression is observed (Figure S3), though we detect an anomalous but probably spurious shallow maximum in the curve that could be associated with the accuracy of our calculations, since differences in *R*(O–H) values are of the order of 10^–4^ Å. Overall, the *R*(O_w_–H) remains practically unaltered in the 0–9 GPa range, whereas the *R*(H···O_S_) decreases to ∼1.60 Å, more than 8%. Additionally, the ∠(O–H···O) slightly bends under increasing pressure from 160° to 156.5°. We conclude that the main pressure-response mechanism of MgSO_4_·1H_2_O is through the reduction of H-bonding, in agreement with our previous polyhedral analysis.

Figure 7 compares the pressure dependence of the H-bond distances in the remaining two representative hydrates: MgSO_4_·4H_2_O (left panel) and MgSO_4_·7H_2_O (right panel). At *p* = 0 GPa, in MgSO_4_·4H_2_O, seven out of eight O–H bonds participate in hydrogen bonding, while one O–H group remains non-hydrogen bonded. This non-H-bonded H is directed toward two oxygen atoms of SO_4_, with the calculated *R*(O···O) distances of 3.054 Å and 3.060 Å at 0 GPa, indicating that they do not belong to the category of hydrogen bonds.^57^ This O–H group gradually forms a conventional H-bond^58^ with one of the sulfate oxygens: an *R*(H···O_S_) value of around 1.90 Å is calculated at 9 GPa (see pink line in Figure 7 (left panel)). The corresponding *R*(O···O) distance decreases from 3.060 to 2.821 Å, while the (∠O–H···O) angle increases from 140° to 155° as pressure increases from 0 to 9 GPa.

**Figure 7:**
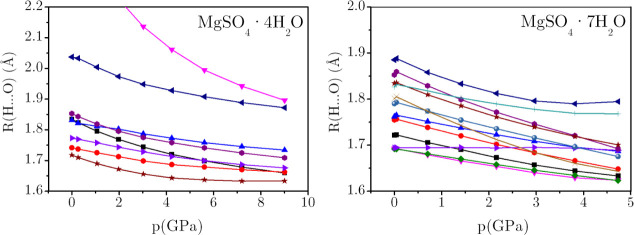
Variations of H-bond distances with pressure for *n* = 4 (left) and 7 (right).

At *p* = 0 GPa, the crystal structure of MgSO_4_·7H_2_O contains 14 hydrogen bonds, involving both O–H···O_S_ and O–H···O_W_, with *R*(H···O) distances ranging from 1.69 to 1.89 Å. Upon compression to 4.5 GPa, these hydrogen bonds become shorter and more isotropically distributed, with distances narrowing to the range of 1.64–1.85 Å. A closer inspection of the distribution reveals clustering into three distinct groups: 1.64–1.69 Å, 1.72–1.75 Å, and 1.80–1.83 Å. This trend reflects the complex network of hydrogen bonding in MgSO_4_·7H_2_O and highlights how pressure selectively compresses and reorganizes the hydrogen bond geometry.

For both MgSO_4_·4H_2_O and MgSO_4_·7H_2_O hydrates, H-bond distances decrease as the pressure rises. The rate and extent of contraction are very similar, noting that the pressure range of the seven-water-molecule case is half that of the four-water-molecule crystal (see Figure 7). This qualitatively similar behavior is consistent with the slightly lower *B*_0_ value of MgSO_4_·7H_2_O that shows a greater number of H-bonds and unit cell volume. Nevertheless, the important point to emphasize is that it is the overall reduction of these bonds that is mainly responsible for the increase in crystal density under pressure.

The pressure-induced changes in intramolecular O–H bond distances and H-bond angles for these hydrates are listed in Figure S4. Under pressure, H-bond angles exhibit diverse behavior with no definite pattern. We also examined the correlation between the covalent *R*(O–H) bond length and the *R*(O···O) hydrogen bond distance as a function of the pressure (not shown in Figure S4). At 0 GPa, O···O–H bonds exhibit an inverse relationship, where a decrease in *R*(O···O) corresponds to a lengthening of *R*(O–H). Previous studies on hydrated MgSO_4_·*n*H_2_O, using both cluster-based calculations^33^ and periodic DFT calculations,^59^ have confirmed the adhesion to established geometric hydrogen bond correlations.^60^ We noticed that this inverse relationship, characteristic of ″normal″ hydrogen bonds, holds up to 9 GPa but could show deviations at higher pressures, indicating potential structural rearrangements or modifications in hydrogen bonding behavior.

## 4 Conclusions

A DFT-based computational investigation of the EOS of MgSO_4_·*n*H_2_O (*n* = 0–11) hydrates was performed to analyze their crystal chemistry under pressure. The aim was to understand compressibility trends as both the degree of hydration *n* and pressure increase. Our results reveal that the bulk modulus decreases as hydration increases, indicating a progressive compressibility along the series. The analysis of polyhedral contributions and S–O, Mg–O, and H-bond length variations with pressure shows that the SO_4_ tetrahedra and MgO_6_ octahedra contribute minimally to the bulk modulus, with more than 90% of the compressibility originated from the water-filled regions. This behavior is driven by the increasing dominance of water-filled spaces and the hydrogen-bond network over the more rigid sulfate and magnesium coordination polyhedra and corresponding chemical bonds. When these hydrates become denser as pressure is applied, it is not because SO_4_ and MgO_6_ reduce their volume. It is the interstitial space, where the H-bond network is acting, that controls the densification mechanism.

A rule of thumb is proposed to estimate the volumes of hydrated salts from just the value of the anhydrous component. It allows us to interpret the structures of the hydrates as formed by an anhydrous framework that always occupies roughly the same space, regardless of the number of water molecules. We also identified two distinct compressibility regimes: one associated with noninterstitial hydration states, where water molecules directly coordinate Mg^2+^, and another governed by interstitial water molecules, which introduce greater structural flexibility.

The findings of this study provide a deeper understanding of the stability and compressibility of hydrated magnesium sulfates. Its calcium analog, CaSO_4_·*n*H_2_O (*n* = 0, 0.5, 2), is a straightforward candidate where our results are expected to provide useful interpretations that could complement studies of this system under hydrostatic and uniaxial stress conditions.^61,62^ By offering comprehensive EOS analysis across multiple hydration states, our work provides a general contribution to future experimental and theoretical studies on the mechanical properties of hydrated salts under extreme conditions. In particular, our results have important implications for planetary science and contribute to the optimization of these materials for thermochemical energy storage applications.

## Supplementary Material



## Notes

Additional computational results including five figures with polyhedral views of unit cells of all the MgSO_4_·*n*H_2_O hydrated salts, evolution of volumes and bulk moduli with *n*, evolution of various structural parameters with pressure, and QTAIM topological analysis of the anhydrous and the MgSO_4_·7H_2_O crystals. One table benchmarking different DFT functionals, and two tables with QTAIM results­(PDF)
