# Supplementary material for: A Chemical Bonding Interpretation of Unusual Compressibility Trends in Hydrated Magnesium Sulfates
Source: Inorg Chem. 2025 Aug 26;64(35):17712–21. doi: 10.1021/acs.inorgchem.5c01765 (PMC12421680; doi:10.1021/acs.inorgchem.5c01765)
Supplement: Supplementary file 1 [file ic5c01765_si_001.pdf]

Supporting Information

A chemical bonding interpretation of unusual compressibility trends in hydrated magnesium sulfates

Getachew G. Kebede[†,‡], Ruth Franco,[‡] Fernando Izquierdo-Ruiz,[¶] Alvaro Lobato,[*,¶] and J. Manuel Recio[*,‡]

†Center for Materials Science and Engineering, Addis Ababa University, P.O. Box 1176, Ethiopia

‡Malta-Consolider Team and Department of Analytical and Physical Chemistry, University of Oviedo, E-33006 Oviedo, Spain

¶Malta-Consolider Team and Departamento de Química Física, Universidad Complutense de Madrid, E-28040 Madrid, Spain

*E-mail: a.lobato@ucm.es; jmrecio@uniovi.es

Table of Contents

Additional Figures and Tables **S3**

Figure S1 .... **S3**

Table S1 .... **S4**

Figure S2 .... **S4**

Figure S3 .... **S5**

Figure S4 .... **S5**

Table S2 .... **S6**

Figure S5 .... **S6**

Table S3 .... **S7**

Authors contributions **S7**

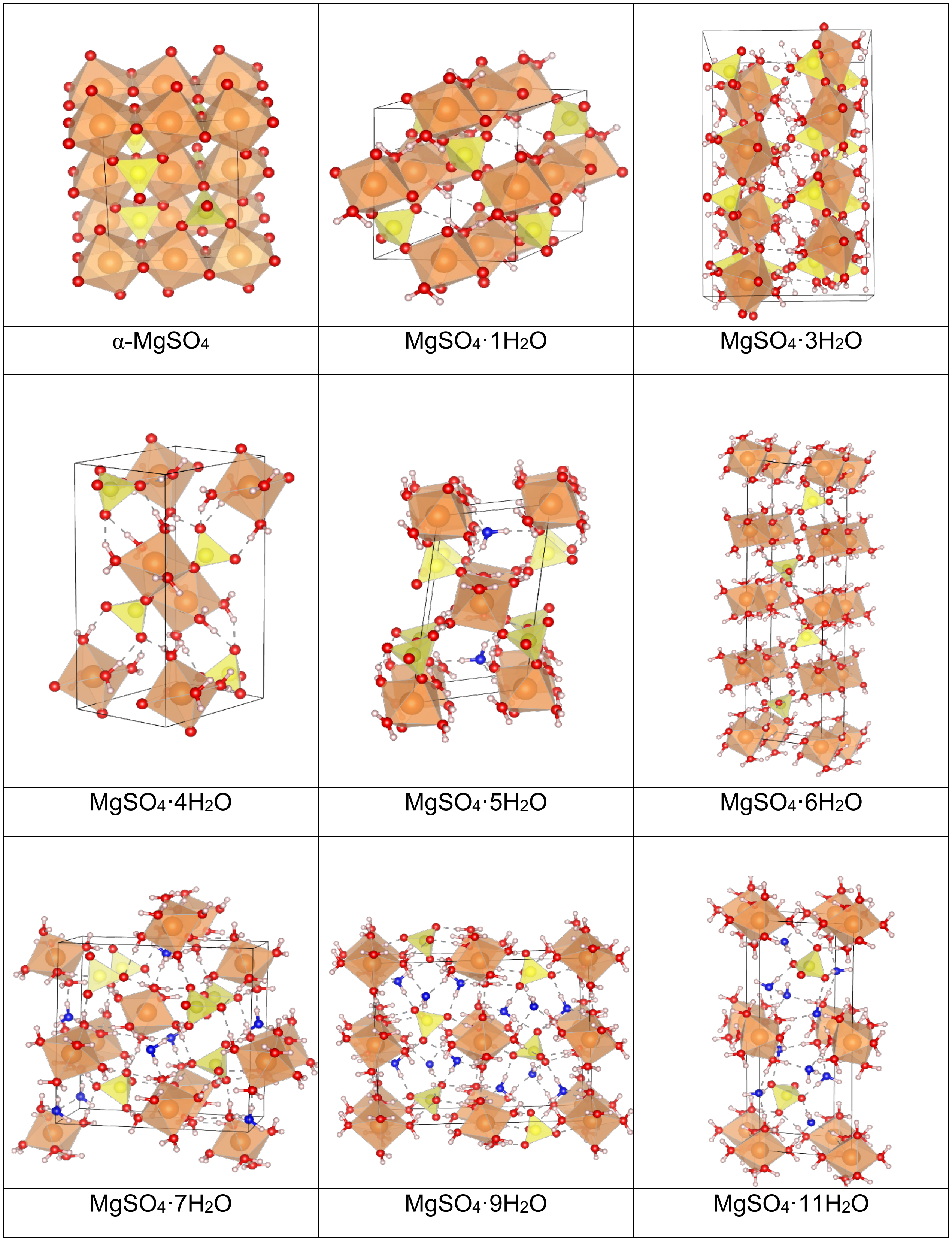

Figure S1. Polyhedral views of all the unit cells of the $MgSO_4{\cdot}nH_2O$ studied in this work. Mg = orange , S = yellow, H = gray, and O = red. Interstitial water oxygen atoms are denoted in blue.

Table S1. Comparison of unit cell parameters (*a, b, c* in Å, and *V* in Å³) of $MgSO_4·nH_2O$ (n = 0–11) computed using four exchange–correlation (*XC*) functionals (PBE, PBE-D3, reV-vdW-DF2, and optPBE-vdW) and benchmarked against experimental crystallographic data. The corresponding experimental references are provided in Table 1 of the main paper.

XC functionals	Unit Cell	$MgSO_4$	$MgSO_4$. $1H_2O$	$MgSO_4$. $4H_2O$	$MgSO_4$. $5H_2O$	$MgSO_4$. $6H_2O$	$MgSO_4$. $7H_2O$	$MgSO_4$. $9H_2O$	$MgSO_4$.1 $1H_2O$
PBE	*V*	278.34	373.87	664.2	376.8	1817.19	997.49	1215.63	722.89
PBE	*a*	5.226	6.802	6.0867	6.292	10.201	12.066	6.807	6.742
PBE	*b*	8.0252	7.948	13.680	10.753	7.230	12.109	12.1419	6.881
PBE	*c*	6.6362	7.766	7.978	6.138	25.063	6.827	14.7546	17.493
PBE-D3	*V*	271.3	361.69	622.98	361.15	1720.19	951.39	1152.48	692.24
PBE-D3	*a*	5.215	6.797	5.868	6.207	9.879	11.939	6.638	6.674
PBE-D3	*b*	7.947	7.815	13.497	10.512	7.295	11.855	11.875	6.719
PBE-D3	*c*	6.547	7.647	7.866	6.078	24.164	6.722	14.628	17.383
reV-vdW-DF2	*V*	268.11	358.52	633.77	362.81	1728.81	956.74	1158.52	697.0
reV-vdW-DF2	*a*	5.213	6.799	5.911	6.218	9.928	11.931	6.669	6.699
reV-vdW-DF2	*b*	7.923	7.808	13.592	10.550	7.284	11.872	11.898	6.734
reV-vdW-DF2	*c*	6.492	7.588	7.889	6.0781	24.201	6.755	14.657	17.414
optPBE-vdW	*V*	273.47	366.29	647.51	371.43	1770.26	980.79	1168.55	713.19
optPBE-vdW	*a*	5.242	6.877	5.951	6.293	10.013	12.022	6.728	6.766
optPBE-vdW	*b*	7.971	7.821	13.691	10.560	7.352	11.969	11.912	6.806
optPBE-vdW	*c*	6.546	7.678	7.947	6.135	24.333	6.816	14.642	17.463
exp	*V*	262.97	355.61	636.78	364.42	1719.08	961.6	1168.55	701.14
exp	*a*	5.169	6.891	5.922	6.314	9.975	11.897	6.728	6.728
exp	*b*	7.868	7.624	13.604	10.565	7.186	11.909	11.925	6.781
exp	*c*	6.467	7.65	7.905	6.030	24.267	6.787	14.642	17.318

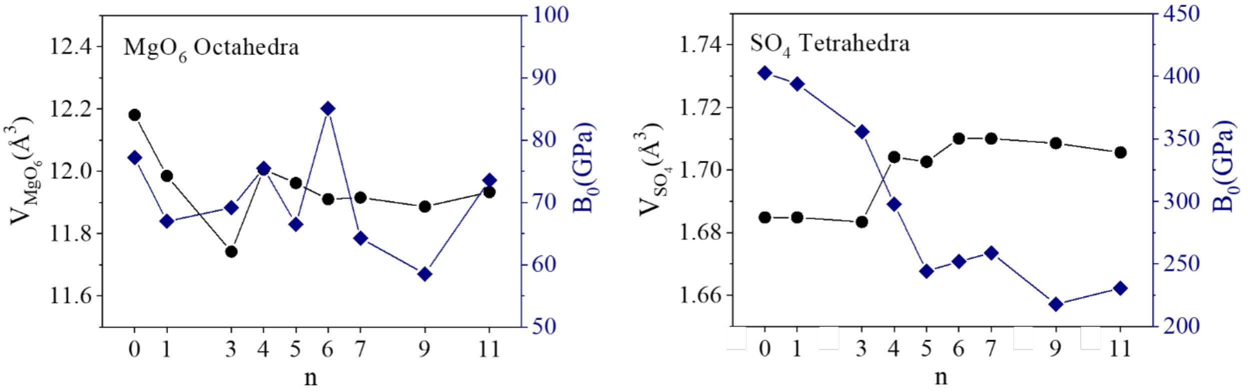

Figure S2. Variations of zero pressure polyhedral volumes, V_0 (black circles), and bulk moduli, B_0 (blue squares), with the number of water molecules contained in the hydrated magnesium-sulfate salts: MgO_6 octahedra (left panel) and SO_4 tetrahedra (right).

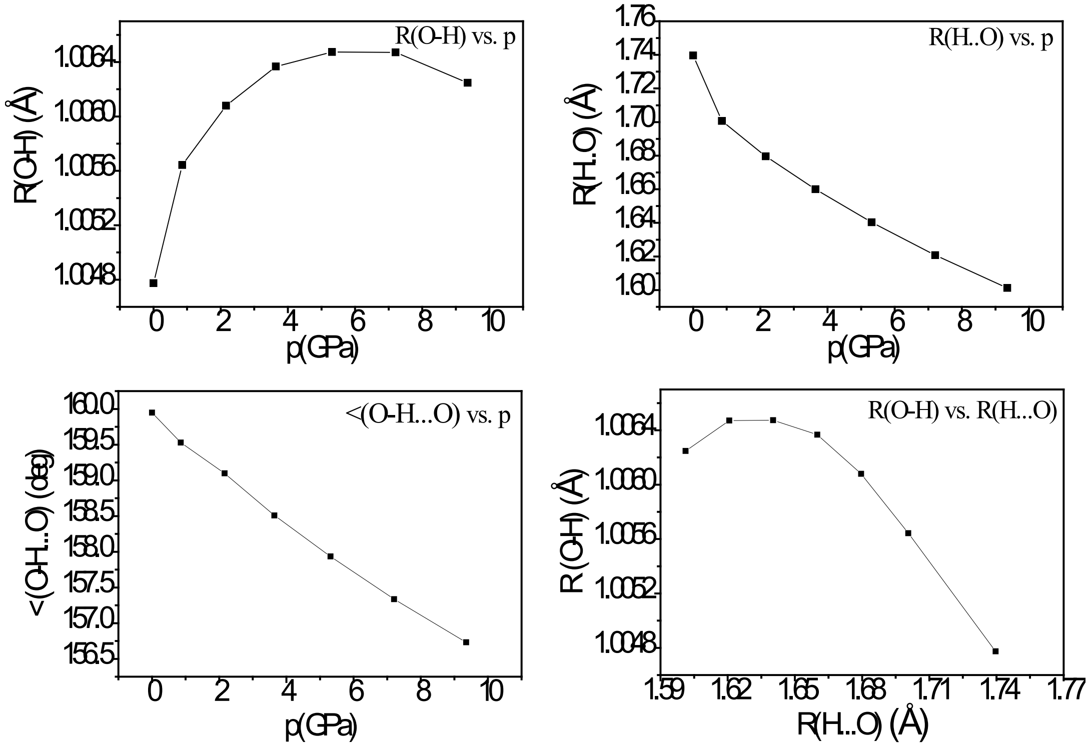

Figure S3. Variations of intramolecular O–H bond distances (R(O-H), hydrogen bond distances (R(H···O)), bond angles (∠(O–H · · · O)), and H-bond correlations with pressure for $MgSO_4 \cdot 1H_2O$.

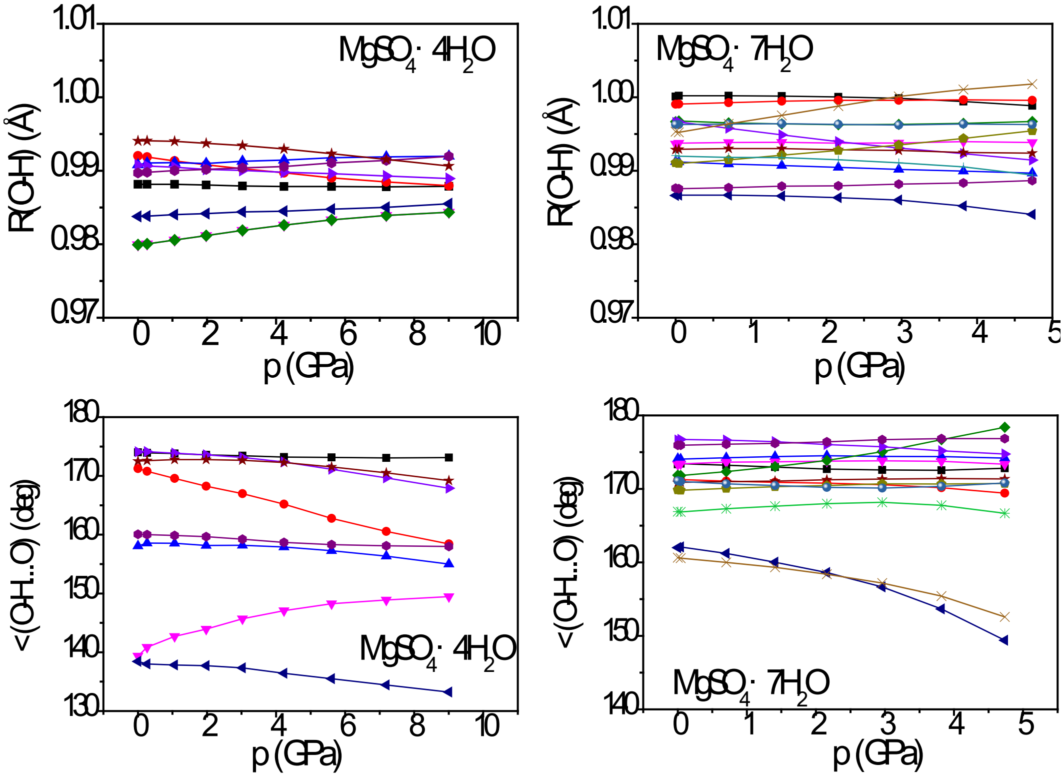

Figure S4. Variations of intramolecular O–H bond distances (upper panels) and H-bond angles (lower panels) as a function of pressure for $MgSO_4 \cdot 4H_2O$ phase (left panels) and $MgSO_4 \cdot 7H_2O$ phase (right panels).

Table S2. Topological analysis according to QTAIM theory. Properties at bond critical points (BCPs) for selected S–O and Mg–O bonds in anhydrous $MgSO_4$. Coordinates refer to the atomic positions at which BCPs were located. The electron density (ρ) and its Laplacian ($\nabla^2\rho$) at the BCPs are given in atomic units.

Bond	Coordinates	Wyckoff position	ρ	$\nabla^2\rho$
S-O (green) Bidentate O	(0.000, 0.690, 0.326)	8f	0.322	0.880
S-O (dark green) Tridentate O	(0.095, 0.602, 0.250)	8g	0.295	-0.818
Mg-O (cyan) Bidentate O	(0.500, 0.390, 0.469)	8f	0.043	0.365
Mg-O (navy) Tridentate O	(0.884, 0.017, 0.106)	16h	0.032	0.194

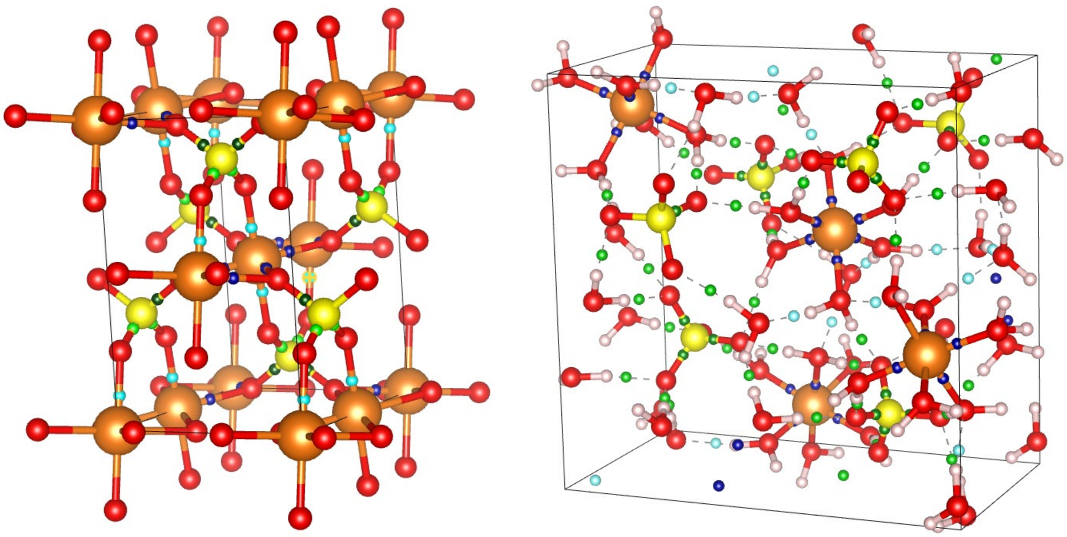

Figure S5. Visualization of bonding environments in MgSO□ (left) and $MgSO_4 \cdot 7H_2O$ (right). Representative bond critical points (BCPs) associated with S–O and Mg–O bonds, and intermolecular hydrogen bonds ($O_w \cdots H$ and $O_S \cdots H$) are depicted, O–H covalent bonds are not represented for clarity. Left image: Blue and dark green spheres denote Mg–O and S-O BCPs for tricoordinated O, respectively. Cyan and light green represents Mg-O and S-O BCPs for bicoordinated O. Right image: dark blue spheres represent Mg-O BCPs, dark green spheres indicates S-O BCPs, while cyan and light green spheres correspond to $O_w \cdots H$ and $O_S \cdots H$ hydrogen bond BCPs, respectively.

Table S3. Bond critical point properties in hydrated MgSO4·7H2O: positions, Wyckoff site, electron density (ρ) and Laplacian(∇^2 ρ) (in a.u.) for S–O, Mg–O, O–H bonds, and intermolecular H-bonds (Ow⋯H and OS⋯H).

Bond	Coordinates	Wyckoff position	ρ	$\nabla^2\rho$
S-O	(0.202, 0.369, 0.531)	4a	0.314	-0.860
S-O	(0.295, 0.683, 0.916)	4a	0.311	0.893
S-O	(0.229, 0.676, 0.007)	4a	0.303	0.898
S-O	(0.202, 0.287, 0.553)	4a	0.295	-0.334
Mg-O	(0.389, 0.077, 0.149)	4a	0.042	0.321
Mg-O	(0.651, 0.638, 0.482)	4a	0.042	0.159
Mg-O	(0.015, 0.922, 0.555)	4a	0.041	0.341
Mg-O	(0.936, 0.363, 0.079)	4a	0.041	0.262
Mg-O	(0.103, 0.958, 0.472)	4a	0.037	0.169
Mg-O	(0.940, 0.331, 0.893)	4a	0.033	0.217
O_w⋯H	(0.944, 0.562, 0.301)	4a	0.046	0.096
O_w⋯H	(0.568, 0.479, 0.051)	4a	0.043	0.086
O_w⋯H	(0.226, 0.036, 0.346)	4a	0.039	0.095
O_w⋯H	(0.981, 0.172, 0.921)	4a	0.038	0.096
O_S⋯H	(0.910, 0.813, 0.227)	4a	0.047	0.116
O_S⋯H	(0.363, 0.559, 0.226)	4a	0.046	0.106
O_S⋯H	(0.717, 0.246, 0.836)	4a	0.045	0.111
O_S⋯H	(0.912, 0.743, 0.798)	4a	0.044	0.090
O_S⋯H	(0.893, 0.207, 0.626)	4a	0.041	0.091
O_S⋯H	(0.713, 0.287, 0.215)	4a	0.039	0.099
O_S⋯H	(0.650, 0.348, 0.479)	4a	0.034	0.090
O_S⋯H	(0.602, 0.012, 0.502)	4a	0.033	0.094
O_S⋯H	(0.106, 0.739, 0.127)	4a	0.031	0.082
O_S⋯H	(0.878, 0.078, 0.509)	4a	0.030	0.073

Author Contributions

G.K and J.M.R. designed the investigation; G.K and R.F performed the calculations; G.K, J.M.R., F.I., and A.L. wrote the original draft of the paper; G.K., R.F., F.I., A.L., and J.M.R. revised and edited the paper.
